# School-Based Interventions on Dental Trauma: A Scoping Review of Empirical Evidence

**DOI:** 10.3390/children10050797

**Published:** 2023-04-28

**Authors:** Kafayat Aminu, Kehinde Kazeem Kanmodi, Jimoh Amzat, Afeez Abolarinwa Salami, Peace Uwambaye

**Affiliations:** 1 Center for Child and Adolescent Mental Health, University College Hospital, Ibadan 200211, Nigeria; bolkaf@yahoo.com; 2 School of Health and Life Sciences, Teesside University, Middlesbrough TS1 3BX, UK; 3 Faculty of Dentistry, University of Puthisastra, Phnom Penh 12211, Cambodia; 4 Cephas Health Research Initiative Inc., Ibadan 200211, Nigeria; donaphice89@gmail.com; 5 Department of Sociology, Usmanu Danfodiyo University, Sokoto 840104, Nigeria; jimoh.amzat@udusok.edu.ng; 6 Department of Sociology, University of Johannesburg, Johannesburg P.O. Box 524, South Africa; 7 Department of Oral and Maxillofacial Surgery, University College Hospital, Ibadan 200211, Nigeria; 8 Department of Preventive and Community Dentistry, School of Dentistry, University of Rwanda, Kigali P.O. Box 4285, Rwanda; upeace1602@gmail.com

**Keywords:** school, dental trauma, intervention, scoping review

## Abstract

Introduction: Accidents involving sports or leisure activities, falls, blows from objects, acts of interpersonal violence, and accidents can all result in dental trauma (also known as traumatic dental injuries [TDIs]). School children are one of the population groups that are highly vulnerable to TDIs. Multiple school-based interventions have been conducted around the world on the prevention and management of TDIs; however, no known scoping review has been conducted to map the evidence. Hence, this study aimed to conduct a scoping review of existing school-based interventions on TDIs. Methods: This scoping review adopted the research design presented by Arksey and O’Malley. Eight research databases—PubMed, SCOPUS, APA PsycINFO, CINAHL Ultimate, AMED (The Allied and Complementary Medicine Database), Child Development & Adolescent Studies, Psychology and Behavioral Sciences Collection, and SPORTDiscuss—were searched to retrieve literature relevant to the scoping review question. Some of the retrieved literature existed in duplicate and was deduplicated using Rayyan software. Twenty papers that fulfilled the inclusion criteria were selected and reviewed. Results: A total of 526 publications were retrieved from the eight databases searched in this scoping review. Ninety-seven publications were duplicates and were removed. After the two-stage screening of the deduplicated copies, only 20 peer-reviewed journal articles were relevant and included in the review. The selected studies sampled a total of 7152 participants whose ages ranged between 9 and 62 years (mean = 10.56 to 46.5; standard deviation = ±0.97 to ±8.1). The findings obtained from the reviewed journal articles revealed that pupils are at high risk of dental trauma and they are more likely to sustain these injuries during school activities. In addition, inadequate understanding, poor attitudes, and low experiential knowledge of dental trauma were found among educators, parents, and even school pupils. The evidence of long-term knowledge retention and a high knowledge score in the experimental groups buttresses the need for continuous first-aid education on dental trauma. Conclusions: The application of multiple educational approaches or modalities in schools will reinforce and promote good first-aid skills and management practices that are essential for preserving traumatized teeth.

## 1. Introduction

The mouth is regarded as the body’s mirror and the window to the body. Quality of life is significantly impacted by oral health; hence, oral health is crucial to overall health [1]. A person’s oral health makes it easier for them to communicate, eat, and socialize without feeling self-conscious or uncomfortable. Even though the burdens of dental caries and periodontal disease have historically been the two biggest global oral health burdens, recent research has shown that trauma to the teeth and jaws is also a major public health issue with an epidemiological component, has numerous clinical and financial implications, and significantly lowers the quality of life at every stage of life [2].

Accidents involving sports or leisure activities, falls, blows from objects, acts of interpersonal violence, and accidents can all result in dental trauma (also known as traumatic dental injuries [TDIs]), as well as injuries to the hard and soft tissues in the oral cavity [3]. Dental trauma exists in various forms, including tooth fractures (which may involve the enamel, cementum, or dentin), tooth abfraction, tooth avulsion, tooth intrusion, tooth luxation, tooth subluxation, tooth intrusion, and tooth extrusion [4,5,6,7,8].

Dental trauma affects 16% to 40% of 6-year-olds in industrialized nations, 4% to 33% of 12- to 14-year-olds, approximately 15% of schoolchildren in various Latin American nations, and about 5% to 12% of 6- to 12-year-olds in the Middle East [9]. It has been reported that the most frequent cause of injuries overall is falling, and children are more likely to have these accidents since their neuromuscular systems are still developing [10]. Due to spending a lot of time engaged in various physical activities, the risk of TDIs is particularly significant among children between the ages of 6 and 12 years [11].

The management of dental trauma begins with first-aid, from the site of the injury, until the point when definitive professional dental care is delivered during dental surgery [8,12]. The clinical management of dental trauma is complex, and severe cases may involve endodontic treatment, dental crowning, periodontal splinting, and tooth replantation [6,7,8,12].

The prognosis and available treatments are greatly influenced by the appropriate steps done as soon as a trauma occurs [8]. First-aid procedures are crucial in cases of serious TDIs (e.g., dental avulsion, dentinal fracture, etc.) because the prognosis is much improved by prompt replantation or careful transportation/immobilization of the tooth to a dental surgeon [8,12]. The approaches for posttraumatic management and prevention should be generally known [3].

Multiple research projects have revealed insufficient knowledge of the first-aid and management of dental trauma; however, the general public, educators, and coaches need to have adequate knowledge of it, and school-based interventions can be used to increase knowledge on dental trauma management [3,11,13,14,15]. In this case, school-based interventions constitute an important oral health improvement strategy for behavioral change that can be implemented following psychological constructs derived from behavioral change models [16].

Several school-based interventions have been conducted on dental trauma prevention and management. However, no known study has conducted a scoping review on these studies to map out the evidence. Therefore, this scoping review aims to compile the existing global evidence in the literature on school-based dental trauma prevention and management.

## 2. Methods

This scoping review adopted the design proposed by Arksey and O’Malley [17] and reported in accordance with the Preferred Reporting Items for Systematic Reviews and Meta-Analyses extension for Scoping Reviews checklist [18].

The scoping review question, “What is the existing empirical evidence on school-based interventions on dental trauma?” was informed by the PCC (P—Population of interest; C—Concept; C—Context) framework, where the populations of interest were teachers, students/pupils, and parents of school children, the concept was interventions on dental trauma, and the context was school settings [19].

On 21 January 2023, a search of relevant literature on the scoping review topic was done using eight research databases—PubMed, SCOPUS, APA PsycINFO, CINAHL Ultimate, AMED (The Allied and Complementary Medicine Database), Child Development & Adolescent Studies, Psychology and Behavioral Sciences Collection, and SPORTDiscuss with Full Text—using a combination of relevant search terms, aided by Boolean operators and truncations, and without year limiters, to retrieve relevant literature published from inception until the specified date (21 January 2023) (Table A1, Table A2 and Table A3 (Appendix A)).

Some of the retrieved literature existed in duplicate and was deduplicated using Rayyan software. Thereafter, the deduplicated copies were screened to exclude literature that was irrelevant to the scoping review. The screening process was two-staged and conducted by two independent reviewers. The first stage involved title and abstract screening while the second stage involved full-text screening.

The following criteria determined if literature was included in the review: refereed journal papers adopting an intervention research design; papers reporting school-based interventions on dental trauma (e.g., flipchart-based interventions, pamphlet-based interventions, leaflet-based interventions, presentation-based interventions, seminar-based interventions, etc.); papers published in English; and papers whose full texts were accessible. However, the following criteria determined if an article was excluded from the review: non-refereed journal papers adopting an intervention research design; papers reporting school-based interventions on issues, not about dental trauma; papers that were not published in English; and papers whose full texts were not accessible.

Only those articles that met the above-specified inclusion criteria were included in the review. Relevant data, such as author names, year of publication, study location (country), study design, study setting (nursery school, primary school or secondary school), study population (teachers, students/pupils, parents), intervention mode (e.g., posters, seminars, etc.), study objectives, findings, and conclusions were extracted from the articles. The extracted data were collated, summarized, and presented in texts and tables.

## 3. Results

**Figure 1:**
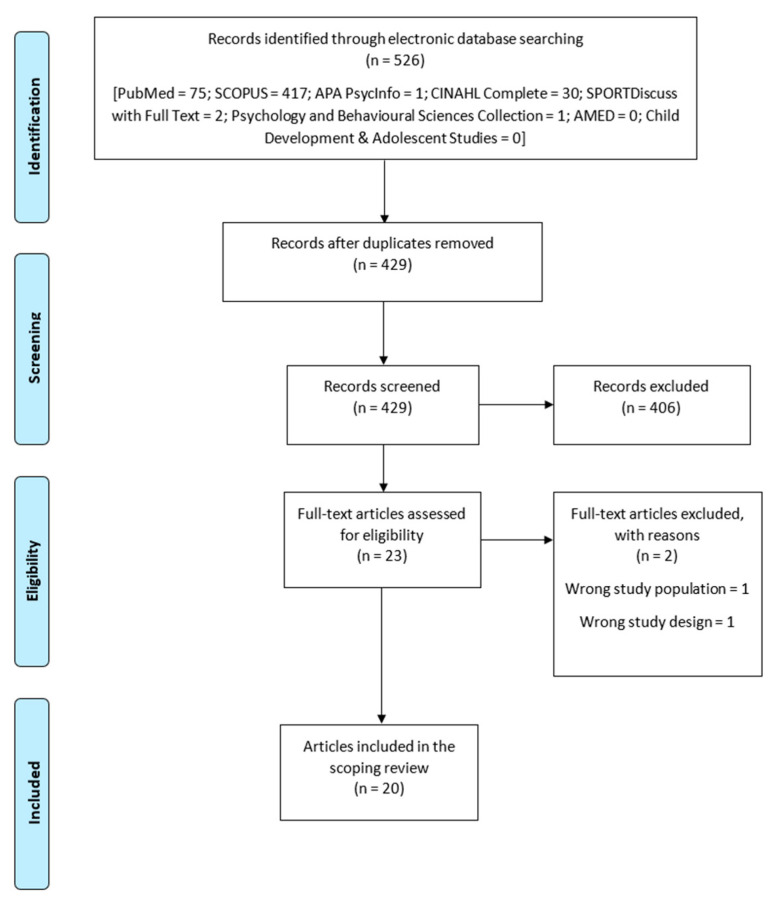
Flow chart diagram.

A total of 526 publications were retrieved from the eight databases searched in this scoping review. Ninety-seven publications were duplicates and were removed. After the two-stage screening of the deduplicated copies, only 20 peer-reviewed journal articles were relevant and included in the review (Figure 1; Table A4 (Appendix A), Table 1). The selected studies sampled a total of 7152 participants whose ages ranged between 9 and 62 years (Mean = 10.56 to 46.5; SD = ±0.97 to ±8.1).

The twenty selected papers were conducted in different parts of the world. One study each was conducted in Syria, Israel, Indonesia, the USA, Hong Kong, and Tanzania. Three studies each were conducted in Brazil, Iran, and Kuwait, and five studies were conducted in India. The population targeted included school pupils (nursery, primary, and secondary), teachers, and parents. The majority introduced interventions (educational); in addition, most authors evaluated the short-term effects of the intervention, whereas only a few assessed the long-term effect of the intervention in the study population. Table 1 provides a summary of the studies.

The results of the review fit into two major themes: baseline knowledge and practices and outcomes of interventions on dental trauma. Each was further classified into sub-topics as presented next.

### 3.1. Baseline Knowledge and Practices for Dental Trauma/Tooth Avulsion

#### 3.1.1. Background Knowledge and Training on Dental Trauma

Background training on dental trauma (DT), including tooth avulsion (TA), was reported in only 3 of the 20 publications. Two focused on schoolteachers in India [23,31] and one on students in Hong Kong [29]. The rate of dental first-aid training was comparatively lower than that of medical first-aid training. About 28.26% and 18.72% (*n* = 214) had received medical first-aid training and dental first-aid training in India [23]. In Hong Kong, 11.40% and 2.10% (*n* = 667), respectively, had such training [29]. The highest rates of medical first-aid training were recorded among rural (87.6%; *n* = 500) and urban (94.8%; *n* = 500) school teachers. The rate of dental first-aid training was also low (rural—8.0%; urban—9.4%) [31]. Additionally, some professionals had received information on TA prior to intervention [26].

#### 3.1.2. Awareness and Understanding of Tooth Avulsion (TA)

The awareness and knowledge of TA were low pre-intervention among different groups [21,24,26]. For all aspects of TA, teachers’ knowledge was inadequate [21,32]. Even among students, baseline knowledge and practice scores were low. Some teachers had higher baseline knowledge than the students [2].

Similarly, the baseline knowledge score was low among intervention and control groups [28] and among intervention groups [11]. Remarkably, the knowledge of the storage medium and the management of avulsed permanent teeth, displaced teeth, and fractured teeth were low among students. More students had a good understanding of the place and time to treat an avulsed tooth and the management of avulsed baby teeth [29]. Some participants understood the appropriate cleaning, handling, and storage of an avulsed tooth. The majority understood the need to reposition a permanent tooth and its urgency and that a milk tooth does not require repositioning (71.9%) [35].

#### 3.1.3. Etiology of Tooth Avulsion

Not many of the reviewed studies scrutinized people’s understanding of DT etiology. Lima et al. reported sports, fights, accidents, and feeding as the aetiologic factors cited by participants in Brazil [24]. Along with accidents linked to sporting activities, Frujeri & Costa [26] documented falls as the major perceived cause of DT. Participants further identified being hit, car accidents, fainting, fighting, as well as occupational hazards as common causes [26]. The majority of teachers (88%) in India mentioned falls as the most common cause of DT among both genders. Sports accidents, ‘kabbadi’ (77%), and fights were also prominent causative factors cited [22].

#### 3.1.4. Population Susceptible to Dental Trauma/Tooth Avulsion

Most teachers in India shared the belief that boys are more susceptible to DT than girls [22].

#### 3.1.5. Emergency Management of Dental Trauma/Avulsed Tooth

At baseline, teachers’ knowledge about the emergency/first-aid management of DT and avulsed and fractured teeth [21,22,23,31,34] was inadequate. Specific knowledge of the replanting, handling, storage medium, and the best time to replant avulsed teeth [21], including permanent and primary avulsed teeth [31], was limited. Most teachers (in urban and rural areas) expressed dissatisfaction with their knowledge/ability to manage dental injuries. Poor management practices and traditional procedures, such as applying turmeric were common among teachers [31].

Teachers’ attitude toward the management of avulsed permanent teeth was likewise poor [23]. However, the majority of educators in another study would refer cases to a professional dentist, a doctor, and a nurse (1.5%) [24]. Similarly, for students, pre-intervention knowledge of the storage of an avulsed tooth [29], appropriate management of an avulsed permanent tooth [20,29], a displaced tooth, and a fractured tooth was deficient [29].

Generally, knowledge of the appropriate storage and/or transportation medium for an avulsed tooth was poor [22,26,29]. Items such as paper towels (58), hydrogen peroxide or alcohol (10), dry gauze (72), hand or pocket (10), plastic bag (26), and ice (54) were cited by participants [26].

#### 3.1.6. Replantation of an Avulsed Tooth

At baseline, knowledge of avulsed tooth replantation varied. Some authors reported a high knowledge [26] while others found limited knowledge [22]. Some professionals also lacked the knowledge of the correct manipulation of an avulsed tooth [26]. However, only a few of them were confident in carrying out the procedure as appropriate. Dental doctors were associated with the management of DT by the majority [26].

The ideal timing for replantation varied among the authors, some stated 30 min to 1 h [23], others stated within 30 min of trauma [20,31] or within 15 min [22]. Before the intervention, only a few participants understood the importance of time in the emergency management of an avulsed tooth [31]. Seven out of every 10 teachers in one study did not understand the ideal time to replant an avulsed tooth prior to intervention [23].

#### 3.1.7. Handling and Cleaning of an Avulsed Tooth

Knowledge of the appropriate method of handling [23] and cleaning the affected tooth before replantation was inadequate as only some participants understood this [22,23,31]. Some professionals would wash the avulsed tooth with a brush or water and soap. Some would wash and store it in hydrogen peroxide or alcohol [26].

#### 3.1.8. Prevention of Dental Trauma

Only one study assessed DT prevention and observed limited awareness and knowledge of DT prevention (use of mouthguards) among schoolteachers [22].

#### 3.1.9. Experiential Knowledge of Dental Trauma

Some teachers had experiential knowledge of DT and avulsed permanent teeth [13]. Teachers from rural areas witnessed more cases of DT during their teaching practice. Conversely, urban teachers managed more cases [31]. Likewise, the majority of teachers in intervention groups (oral presentation group—52.2% and educational leaflet group—51.7%) and some mothers (17.6%) had witnessed DT in their students and children, respectively [11]. Some participants had previously provided emergency first-aid care for TA (18.37%) and other DT (26.69%) cases [26]. Further, the mean self-reported practice score was higher in some groups (educational leaflet) than in others (oral presentation), (3.67 ± 1.97 vs. 3.26 ± 2.12) [11].

Although Sedlaceck et al. did not assess baseline knowledge, they found that many participants (40.5%) had experienced some forms of DT, and the majority (62.8%) understood TA [25].

#### 3.1.10. Determinants of Baseline Knowledge and Practices

Many authors failed to assess the effect of previous training on baseline knowledge and practices, although some participants claimed they received medical and dental first-aid training. Taranath et al. however, revealed that the previous training did not impact the knowledge of DT management before the intervention [23]. Educational qualification was statistically associated with the mean knowledge score nevertheless, and no demographic factor had a similar effect on the self-reported practice score [11].

### 3.2. Outcomes of Interventions on Dental Trauma/Tooth Avulsion

#### 3.2.1. Post-Intervention Knowledge and Practices for Tooth Avulsion

For the majority of the reviewed publications, the intervention produced desired outcomes in the study population as the knowledge level significantly improved from pre-intervention to post-intervention [11,20,23,24,26,29,30,32,34,35]. This was evident in most knowledge topics measured: understanding of TA, extra-alveolar time [24,25,32], permanent and primary TA, [25,32], replantation and cleaning of avulsed teeth, and suitable storage/transportation medium for an avulsed tooth [13,21,23,24,25,26,31,32,35].

Knowledge of avulsed tooth management [31], handling [23,24], first-aid care [13,20,21,25], actions to take in case of TA [25,31], reattachment of a broken tooth part, and the importance of avulsed tooth replantation [11] also improved. Similarly, the knowledge that anyone can replant an avulsed tooth changed positively [25].

In another study, the diverse intervention strategies introduced failed to produce significant improvement in the knowledge [24]. Where knowledge improved significantly [11,25,27,28,30], and on topics for which knowledge change was insignificant [24], some differences were conspicuous among the assessed groups. Most interventions improved knowledge better among the experimental group(s) than the control group [11,25,27,28,29,32] and in some experimental groups than others [27,28,30,35].

Moreover, a low post-intervention knowledge score was reported by some authors (although a significant improvement on certain topics was remarkable) [13]. Moreover, for certain topics, the post-intervention knowledge failed to improve or declined [2,28,29], or the improvement was below the expected levels [13,31].

The immediate post-intervention knowledge improved better than the long-term knowledge for most topics assessed [11,34]. The long-term knowledge scores were lower for such topics as storing tooth fragments, cleaning a fallen tooth, and the ideal time for replanting an avulsed tooth [11,34]. Nevertheless, over time, some knowledge improvement and retention were evident as participants’ understanding of specific topics improved significantly [11,34].

#### 3.2.2. Change in Attitude and Management Practices for Tooth Avulsion

For participants who witnessed [30] or experienced [33] cases of TA post-intervention and those who did not, some positive change in the emergency care practices was manifested [30,33]. Examples of a positive change in actions included looking for the tooth, seeking professional help within 30 min [20] until one to six hours after avulsion, an increase in the number of clinic visits [33], confidence in the ability to replant an avulsed tooth, association of dental doctor to DT management, physical examination [26], and a positive attitude to professional referral [24].

Some unimpressive post-intervention attitudes included not storing/transporting avulsed teeth in a recommended storage medium, late clinical presentation after dental trauma, and poor self-care actions [33].

#### 3.2.3. Determinants of Intervention Outcomes

The time of post-intervention assessment played a major role in the outcome of intervention among some participants as knowledge and practice scores increased immediately after the intervention and then decreased 3 months later. Likewise, 1 week after the first intervention, the students’ mean practices score was higher in the slideshow than in the flip chart group [2]. Contrariwise, in another study, teachers’ knowledge and practice scores increased immediately and 3 months later [2].

The type or nature of the intervention likewise influenced the knowledge change following the intervention. Among teachers, the mean practice score significantly improved in the flipchart group than in the slideshow group [2]. The knowledge of those in the mobile applications group was comparatively better than that in the lecture group and the group where the two methods were combined [35].

Demographic characteristics such as age, work experience, employment status, and educational level failed to influence teachers’ knowledge scores. Demographic factors also had no association with teachers’ self-reported practice scores [11]. Another study, however, revealed that teachers’ age determined the overall change in knowledge and for specifics such as the general knowledge of DT, emergency care for tooth injuries, extraoral time, washing of avulsed teeth, and the storage medium. The teachers’ income level also determined their knowledge, attitude, and practice (KAP) scores [30]. Teachers’ self-reported practice scores had an insignificant relationship with post-intervention knowledge at 1 month and 6 months [11].

Furthermore, parents’ age, educational level, and income were associated with their KAP scores [30]. Similarly, the maternal knowledge score was determined by age and home ownership status. The self-reported practice was significantly influenced by educational status and home ownership [27].

Knowledge and self-reported practice for a dental injury were significantly related [27]. In addition, some changes in the control group’s knowledge were attributed to the effects of contaminants such as interactions with teachers in the experimental group, the display of posters, the distribution of circulars to teachers, the visitation of children, and the involvement of major stakeholders [13].

## 4. Discussion

We reviewed 20 publications on dental trauma/tooth avulsion. Different forms of educational interventions were adopted in the studies. The intervention modality ranged from an oral presentation, lecture, question-and-answer sessions and informational posters, leaflets, flipcharts, mailed guidelines/brochures, interactive seminars, notebooks with figures and informative texts, and animated videos to smartphone applications. A combination of the intervention approaches was adopted in some of the studies reviewed while a single approach was likewise utilized by some authors. Only one study reported clinical experiments [21]. All interventions were delivered by experts, mostly dentists. However, it was not reported to what extent the multiple intervention approaches determined the outcome.

The publications reviewed covered various topics on the knowledge of, the practices of, and the attitudes to dental trauma and tooth avulsion. Across the studies, more individuals in the experimental group(s) experienced a significant knowledge increase, although the level of increase varied. Only a few studies recorded a knowledge decline, stagnation, or insignificant improvement despite the intervention [2,28]. Some progress was also recorded in the control groups, which a group of researchers attributed to the contamination effects [13]. Other authors did not report factors responsible for the control groups’ knowledge change.

The current review found poor baseline knowledge of emergency dental care, as the knowledge score was significantly lower than the post-intervention score in most of the studies, except for instances where participants’ knowledge of specific topics was adequate before partaking in the intervention. This conforms with the findings of a systematic review that revealed inadequate knowledge of dental trauma management [36]. Our review shows that the intervention introduced on the subject helped to improve participants’ knowledge of avulsed tooth management and emergency management practice, including handling, cleaning, and means of storage and transportation.

The duration between the intervention and post-test assessment may have influenced the observed intervention outcomes. Some of the reviewed studies performed the initial or the only post-intervention assessment as soon as the intervention was concluded, while some did so one week later or two weeks following the intervention. Additionally, some authors assessed the intervention at three or six months, and some did so as long as 36 months afterward. The knowledge score for the early post-test was higher than the knowledge score after a few months. Nevertheless, some evidence of knowledge retention was observed in a longitudinal study among Iranian teachers who successfully recalled information received on dental trauma even after a period of three years [34].

Furthermore, a few of the studies (3 of the 20 publications) in this review found evidence of background training and experiential knowledge of dental trauma and tooth avulsion. Compared to medical first-aid training, the rate of dental first-aid training was seemingly low. Having previous training in dental trauma was not, however, significantly impactful on the baseline knowledge or practice. The after-effect of experiential knowledge on the baseline and post-intervention knowledge and experience was not explored by the authors.

The majority of the studies reviewed sampled schoolteachers, three studies focused on parents, three sampled students, some combined populations of teachers and pupils, and some interviewed the three groups (pupils, parents, and teachers), while others focused on subject teachers (physical and health education) and other professionals (elementary school teachers, physical education professionals, bank employees, dental doctors, and pediatricians). Where the three groups participated in the same study, teachers’ performance in the emergency management of tooth avulsion was significantly better than that of students and parents. A major fact worthy of note is that although most of the study groups represent stakeholders, physical and health education teachers are often more likely to be approached for emergency dental care than other teachers or educators within the school setting.

All of the studies except one adopted a survey method to measure the participants’ knowledge of dental trauma and tooth avulsion before and after the intervention; the outlier study combined both quantitative and qualitative approaches for the assessment [24]. Hence, this may have impacted the study findings.

The studies that assessed the intervention’s impact on the practical application of knowledge acquired through the intervention revealed that while the rate of clinic visits for tooth avulsion increased post-intervention, the intervention failed to change self-care actions taken by some participants. Only a few participants were confident of carrying out avulsed tooth replantation effectively.

Not many of the reviewed studies scrutinized people’s understanding of the etiology and prevention of dental trauma. The only study that assessed participants’ knowledge of the population at risk of dental trauma shared that boys are believed to be more susceptible to dental trauma than girls [22]. Other studies have documented that boys are twice as likely to experience dental trauma than girls [37,38]. These and other epidemiological features are germane to dental avulsion prevention, nonetheless, they were not adequately explored in the reviewed publications.

This review acknowledges that school-based educational interventions are important for behavioral change. However, the school setting is a closed environment for a particular population who is likely to internalize the educational intervention as if it is part of the learning routines. This must have significantly influenced the post-intervention differences. This constitutes a limitation when applying the same intervention to the general population who might not see the intervention as something that should be routinely internalized. Such intervention might require a longer duration to be internalized among the general population. The observed experiential knowledge might also be due to common incidents of dental trauma among school children.

## 5. Conclusions

Pupils are at high risk of physical injuries, accidents, and dental trauma, and they are more likely to sustain these injuries during school activities. However, this review established inadequate understanding, poor attitudes, and a low experiential knowledge of dental trauma and tooth avulsion among educators, parents, and even school pupils, especially at the pre-intervention stage. Prompt management of tooth avulsion and other forms of dental trauma by educators is essential for good prognosis, tooth loss reduction, improvement of treatment outcomes, and general quality of life. The reviewed school-based intervention modalities improved the knowledge, attitude, and practices in the study population. This indicates that educational activities targeting the prevention and management of dental injuries in children are likely to enhance positive treatment outcomes. Further, if such interventions are targeted at stakeholders and introduced at regular intervals, a significant increase in the knowledge of avulsed tooth handling, the storage and transportation medium, and timely help-seeking among others will forestall tooth loss and promote a good quality of life of school pupils.

## Figures and Tables

**Table 1:** Summary of Reviewed Publications.

S/N	Authors and Year	Country	Study Design	School Type	Study Population	Injury Type	Mode of Intervention	Time of Post-Test Assessment	Sample Size
01	Zaher and Dashash (2021) [20]	Syria	Interventional educational study	Primary schools	School children (9–12 years)	Avulsion of permanent teeth	Educational poster	2 months following the intervention	550
02	Holan et. al. (2006) [13]	Israel	Not indicated	Primary	Physical education teachers	Dental trauma	Educational seminar	2 months before and 10 months after the intervention	226
03	Katthika, Fauziah and Budiardjo (2020) [21]	Indonesia	Clinical experimental study	Primary	Public elementary school teachers	Dental avulsion	Animated video	Directly after the animated video was presented	54
04	Karande et al. (2012) [22]	Pune, India	Not indicated	Primary and secondary (public and private)	School teachers of both primary and secondary sections of private and government-aided schools	Dentoalveolar traumatic injuries	Lectures on emergency management of dentoalveolar injuries	3 months after intervention	216
05	Taranath, Senaikarasi, and Manchanda (2017) [23]	India	Not indicated	Primary school	Primary school teachers	Traumatized avulsed teeth	A health educational tool	3 months	214
06	Lima et al. (2021) [24]	Brazil	Exploratory intervention study	Elementary school	Elementary school teachers (197) and pedagogical coordinators (24)	Dental avulsion	Conventional education and problematizing education	30 days	221 (197 teachers and 24 pedagogical coordinators)
07	Sedlaceck et al. (2022) [25]	Brazil	Intervention study	Primary school	Sixth-grade pupils (11 to 12 years)	Dental avulsion	Notebooks with figures and informative texts about dental avulsion and replantation printed on the covers	9 months	242
08	Frujeri and Costa (2009) [26]	Brazil	Not stated	Not applicable	Groups of professionals (elementary school teachers, physical education professionals, bank employees, dental doctors, and pediatricians)	Tooth avulsion	Lecture containing texts and images obtained from books and real case reports	2 months after the lecture	479
09	Razeghi et al. (2019) [11]	Iran	Interventional study	Primary	Primary school teachers	Traumatic dental injuries	Educational leaflet and oral presentation whose contents were prepared based on the “Save Your Tooth” poster (IADT 2011)	1 and 6 months after the interventions	453
10	Razeghi et al. (2020) [27]	Iran	Longitudinal interventional study	Public elementary schools	Mothers of 8-year-old children	Traumatic dental injuries	Poster and pamphlet	3 months after the interventions	201
11	McIntyr et al. (2008) [28]	USA	Longitudinal, time-series research design	Public elementary schools	Elementary school staff	Traumatic dental injuries	Pamphlets and lecture	Immediately after and 3 months after the intervention	111
12	Young, Wong, and Cheung (2014) [29]	Hong Kong	Cluster randomized controlled trial	Secondary Schools	Secondary school Students	Dental trauma	Educational poster	2 weeks	667
13	Srilatha, Shekar, and Krupa (2021) [2]	India	Interventional study	Secondary	Students and teachers	Dental trauma and tooth avulsion	Health education with audiovisual aids (flip charts, slideshow)	1 week and 3 months	1234 (1180 students and 54 teachers)
14	Grewal, Shangdiar, and Samita (2015) [30]	Amritsar city	Randomized/cross-sectional study design	Intermediate school	Parents, teachers, and school children	Avulsed permanent teeth	Flip cards and posters in the local language of the area and English; slide presentations and a interactive seminars/lectures	3 months	589
15	Pujita et al. (2013) [31]	India	Prospective intervention study	Primary and secondary schools	Teachers	Dental trauma	Lecture using Power Point presentation or self-prepared manual (flipchart) containing pictures	3 months	1000
16	Al-Asfour, Andersson, and Al-Jam (2008) [32]	Kuwait	Not stated	Intermediate school	Intermediate school teachers	Avulsed teeth	Lecture	6 months	74
17	Al-Asfour and Andersson (2008) [32]	Kuwait	Not stated	Primary school	Parents	Tooth avulsion	Information leaflets	1 week	150
18	Kahabuka (2001) [33]	Dar es Salam, Tanzania	Not stated	Nursery; Primary school	School teachers/pupils	Dental trauma	Seminar and mailed guidelines/brochure	5 months before and 6 months after the intervention	346 (156 teachers; 190 children)
19	Raoof et al. (2014) [34]	Iran	Longitudinal, time series, and self-control		Health teachers	Dental trauma	Oral presentation, question-and-answer sessions and informational posters	Immediately after and 36 months after the intervention	38
20	Al-Musawi, Al-Sane, and Andersson, (2017) [35]	Kuwait	Not stated	Elementary and intermediate school	Elementary and intermediate school teachers	Tooth avulsion	Lecture; lecture and smartphone app (Dental Trauma App),	Immediately after the intervention	87

## Data Availability

Data sharing is not applicable to this article as no new data were created or analyzed in this study. Kehinde Kazeem Kanmodi had full access to all of the data in this study and takes complete responsibility for the integrity of the data and the accuracy of the data analysis. **Ethical Considerations:** Being a scoping review, ethical approval is not applicable to this study, as this study did not collect data from human or animal subjects but from an open research repository.

## Appendix A

**Table A1:** Search string for the PubMed database search.

Tag	Subject Search	Search String
#1	Dental injury	((((((((((((((((((((root fracture[Title/Abstract]) OR (dental fracture[Title/Abstract])) OR (tooth fracture[Title/Abstract])) OR (enamel fracture[Title/Abstract])) OR (dentin* fracture[Title/Abstract])) OR (dental abfraction[Title/Abstract])) OR (tooth abfraction[Title/Abstract])) OR (dental avulsion[Title/Abstract])) OR (tooth avulsion[Title/Abstract])) OR (traumatic dental injur*[Title/Abstract])) OR (crown fracture[Title/Abstract])) OR (dental concussion[Title/Abstract])) OR (tooth concussion[Title/Abstract])) OR (dental luxation[Title/Abstract])) OR (tooth luxation[Title/Abstract])) OR (dental subluxation[Title/Abstract])) OR (tooth subluxation[Title/Abstract])) OR (dental intrusion[Title/Abstract])) OR (tooth intrusion[Title/Abstract])) OR (dental extrusion[Title/Abstract])) OR (tooth extrusion[Title/Abstract])
#2	Intervention	((((intervention[Title/Abstract]) OR (program*[Title/Abstract])) OR (trial[Title/Abstract])) OR (experiment*[Title/Abstract])) OR (quasi-experiment*[Title/Abstract])
#3	School	(((((((((((secondary school[Title/Abstract]) OR (high school[Title/Abstract])) OR (middle school[Title/Abstract])) OR (elementary school[Title/Abstract])) OR (primary school[Title/Abstract])) OR (teacher[Title/Abstract])) OR (student[Title/Abstract])) OR (pupil[Title/Abstract])) OR (classroom[Title/Abstract])) OR (school[Title/Abstract])) OR (nursery[Title/Abstract])) OR (kindergarten[Title/Abstract])
#4	#1 AND #2 AND #3	(#1) AND (#2) AND (#3)

**Table A2:** Search string for the SCOPUS database search.

Tag	Subject Search	Search String
#1	Dental injury	(TITLE-ABS-KEY (root AND fracture) OR TITLE-ABS-KEY (dental AND fracture) OR TITLE-ABS-KEY (tooth AND fracture) OR TITLE-ABS-KEY (enamel AND fracture) OR TITLE-ABS-KEY (dentin* AND fracture) OR TITLE-ABS-KEY (dental AND abfraction) OR TITLE-ABS-KEY (tooth AND abfraction) OR TITLE-ABS-KEY (dental AND avulsion) OR TITLE-ABS-KEY (tooth AND avulsion) OR TITLE-ABS-KEY (traumatic AND dental AND injur*) OR TITLE-ABS-KEY (crown AND fracture) OR TITLE-ABS-KEY (dental AND concussion) OR TITLE-ABS-KEY (tooth AND concussion) OR TITLE-ABS-KEY (dental AND luxation) OR TITLE-ABS-KEY (tooth AND luxation) OR TITLE-ABS-KEY (dental AND subluxation) OR TITLE-ABS-KEY (tooth AND subluxation) OR TITLE-ABS-KEY (dental AND intrusion) OR TITLE-ABS-KEY (tooth AND intrusion) OR TITLE-ABS-KEY (dental AND extrusion) OR TITLE-ABS-KEY (tooth AND extrusion))
#2	Intervention	(TITLE-ABS-KEY (intervention) OR TITLE-ABS-KEY (program*) OR TITLE-ABS-KEY (trial) OR TITLE-ABS-KEY (experiment*) OR TITLE-ABS-KEY (quasi-experiment*))
#3	School	(TITLE-ABS-KEY (secondary AND school) OR TITLE-ABS-KEY (high AND school) OR TITLE-ABS-KEY (middle AND school) OR TITLE-ABS-KEY (elementary AND school) OR TITLE-ABS-KEY (primary AND school) OR TITLE-ABS-KEY (teacher) OR TITLE-ABS-KEY (student) OR TITLE-ABS-KEY (pupil) OR TITLE-ABS-KEY (classroom) OR TITLE-ABS-KEY (school) OR TITLE-ABS-KEY (nursery) OR TITLE-ABS-KEY (kindergarten))
#4	#1 AND #2 AND #3	(#1) AND (#2) AND (#3)

**Table A3:** String for the search of other databases (CINAHL Ultimate, AMED (The Allied and Complementary Medicine Database), Child Development & Adolescent Studies, Psychology and Behavioral Sciences Collection, SPORTDiscuss with Full Text, and APA PsycInfo) via the EBSCO interface.

Tag	Subject Search	Search String
S1	Dental injury	AB root fracture OR AB dental fracture OR AB tooth fracture OR AB enamel fracture OR AB dentin* fracture OR AB dental abfraction OR AB tooth abfraction OR AB dental avulsion OR AB tooth avulsion OR AB traumatic dental injur* OR AB crown fracture OR AB dental concussion OR AB tooth concussion OR AB dental luxation OR AB tooth luxation OR AB dental subluxation OR AB tooth subluxation OR AB dental intrusion OR AB tooth intrusion OR AB dental extrusion OR AB tooth extrusion
S2	Intervention	AB intervention OR AB program* OR AB trial OR AB experiment* OR AB quasi-experiment*
S3	School	AB secondary school OR AB high school OR AB middle school OR AB elementary school OR AB primary school OR AB teacher OR AB student OR AB pupil OR AB classroom OR AB school OR AB nursery OR AB kindergarten
S4	S1 AND S2 AND S3	(S1) AND (S2) AND (S3)

**Table A4:** List of the literature considered for full text screening and the screening outcomes.

S/N	Refs.	Screening Outcomes	
		Include	Exclude
1	[25]	Yes	
2	[24]	Yes	
3	[28]	Yes	
4	[35]	Yes	
5	[39]	Yes	
6	[40]	Yes	
7	[22]	Yes	
8	[30]	Yes	
9	[11]	Yes	
10	[26]	Yes	
11	[31]	Yes	
12	[27]	Yes	
13	[41]		Yes (Wrong study population)
14	[20]	Yes	
15	[2]	Yes	
16	[21]	Yes	
17	[42]		Yes (Wrong study design)
18	[23]	Yes	
19	[29]	Yes	
20	[34]	Yes	
21	[32]	Yes	
22	[13]	Yes	
23	[33]	Yes	
